# α-Synuclein oligomers mediate the aberrant form of spike-induced calcium release from IP_3_ receptor

**DOI:** 10.1038/s41598-019-52135-3

**Published:** 2019-11-04

**Authors:** Kenji Yamamoto, Yasuhiko Izumi, Monami Arifuku, Toshiaki Kume, Hideyuki Sawada

**Affiliations:** 1 Department of Neurology and Clinical Research Center, National Hospital Organization Utano National Hospital, Kyoto, Japan; 2 Laboratory of Pharmacology, Kobe Pharmaceutical University, Kobe, Japan; 3 Department of Pharmacology, Graduate School of Pharmaceutical Sciences, Kyoto University, Kyoto, Japan; 4 Department of Applied Pharmacology, Graduate School of Medicine and Pharmaceutical Sciences, University of Toyama, Toyama, Japan

**Keywords:** Dementia, Parkinson's disease, Dementia, Parkinson's disease

## Abstract

Emerging evidence implicates α-synuclein oligomers as potential culprits in the pathogenesis of Lewy body disease (LBD). Soluble oligomeric α-synuclein accumulation in cytoplasm is believed to modify neuronal activities and intraneural Ca^2+^ dynamics, which augment the metabolic burden in central neurons vulnerable to LBD, although this hypothesis remains to be fully tested. We evaluated how intracellular α-synuclein oligomers affect the neuronal excitabilities and Ca^2+^ dynamics of pyramidal neurons in neocortical slices from mice. Intracellular application of α-synuclein containing stable higher-order oligomers (αSNo) significantly reduced spike frequency during current injection, elongated the duration of spike afterhyperpolarization (AHP), and enlarged AHP current charge in comparison with that of α-synuclein without higher-order oligomers. This αSNo-mediated alteration was triggered by spike-induced Ca^2+^ release from inositol trisphosphate receptors (IP_3_R) functionally coupled with L-type Ca^2+^ channels and SK-type K^+^ channels. Further electrophysiological and immunochemical observations revealed that α-synuclein oligomers greater than 100 kDa were directly associated with calcium-binding protein 1, which is responsible for regulating IP_3_R gating. They also block Ca^2+^-dependent inactivation of IP_3_R, and trigger Ca^2+^-induced Ca^2+^ release from IP_3_R during multiple spikes. This aberrant machinery may result in intraneural Ca^2+^ dyshomeostasis and may be the molecular basis for the vulnerability of neurons in LBD brains.

## Introduction

A growing body of evidence implicates α-synuclein oligomers are potential culprits in the pathogenesis of Lewy body dementia (LBD), which refers to dementia with Lewy bodies and Parkinson’s disease with dementia^1^. The presence of α-synuclein oligomers has been demonstrated in LBD brains^2,3^, not only in the neuropil, but also in the soma of LBD vulnerable neurons^4^. The aggregation of α-synuclein is upregulated by either mutations to α-synuclein or exposure to dopamine^1^. α-Synuclein oligomers mediate toxicity that occurs via several intracellular mechanism such as mitochondrial and endoplasmic reticulum (ER) stress and an impaired autophagy-lysosomal pathway^5,6^. Therefore, the α-synuclein oligomer is a key molecule in respect to the toxicity to LBD vulnerable neurons.

Dysregulated Ca^2+^ homeostasis has emerged as an underlying pathological mechanism in LBD; it triggers the formation of α-synuclein oligomers, mitochondrial and ER stress, and the inhibition of autophagy and lysosomal pathways, thereby prompting neurodegeneration^5,7^. Epidemiological studies indicate that L-type VDCC (L-VDCC) blockers diminish the risk of Parkinson’s disease (PD)^8,9^. In general, LBD vulnerable neurons such as neurons in the substantia nigra pars compacta (SNc), locus coeruleus, raphe nuclei and the nucleus basalis of Meynert, have a common physiological phenotype; an autonomous pacemaker, broad and slow spiking, or lower expression of Ca^2+^-binding proteins. These physiological characteristics lead to increased cytosolic Ca^2+^ and augment the metabolic burden in these neurons critical for selective neuronal degeneration^7^. In LBD, Lewy bodies appear in neocortical pyramidal neurons and contribute to dementia^10,11^. This raises a question on how intraneural oligomeric α-synuclein can pathologically modify neuronal activity and intracellular Ca^2+^ dynamics in neocortical neurons; this question remains to be answered.

We previously demonstrated how Ca^2+^ or K^+^ channels are involved in the regulation or pathophysiological alteration of neocortical pyramidal cell excitability and Ca^2+^ dynamics. This was performed by using intracellular injection of bioactive molecules or proteins such as inositol trisphosphate (IP_3_), homer1a and amyloid-β through a patch pipette^12–17^, and the results obtained by these methods were compatible with those observed in neurons having physiologically produced IP_3_ or homer1a proteins in cytoplasm, or in neurons of 3xTg Alzheimer’s disease model mice^12–17^. By applying the same methodology, the present study aimed to elucidate the effects and mechanisms of intracellular α-synuclein oligomers on neuronal excitabilities and Ca^2+^ dynamics, by introducing α-synuclein protein into pyramidal neurons in cortical slices from mice.

## Results

### Intracellular application of α-synuclein oligomers reduces spike frequency by enhancing AHP during multiple spikes in neocortical neurons

**Figure 1:**
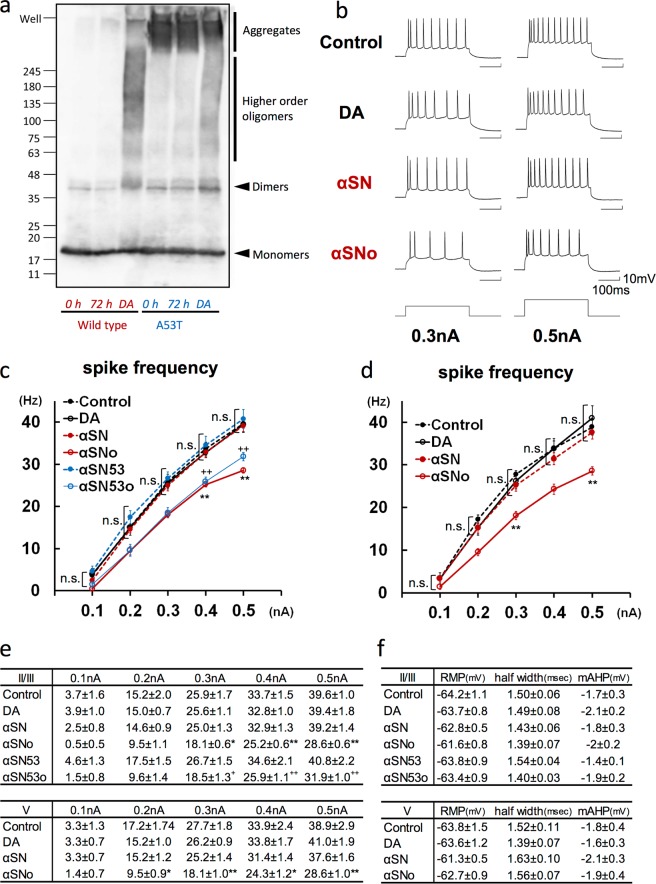
Intracellular application of αSNo reduced spike frequency during depolarizing current injection. (**a**) α-Synuclein oligomerization in the presence of dopamine. Wild-type and A53T variant forms of α-synuclein were incubated for 72 h with dopamine (*DA*) or without dopamine (72 *h*) and compared with a sample under no incubation (0 *h*). Both wild-type and A53T α-synuclein formed oligomers only in the presence of dopamine. (**b**) Specimen recordings of action potentials during positive current pulses (300 ms, 0.3 nA and 0.5 A) in neurons injected by vehicle solution (Control), dopamine incubated without α-synuclein (DA), α-synuclein incubated without dopamine (αSN), and α-synuclein incubated with dopamine (αSNo). The interspike interval was prolonged and the spike frequency was reduced in αSNo-administered neurons. Calibration: 100 ms, 10 mV. (**c**) Average spike frequencies elicited by varying positive current steps (0.1–0.5 nA) in neurons of layer II/III in the frontal cortex. The frequency was significantly lower in neurons infused with αSNo (*<0.05, **<0.01, One way ANOVA) or A53T α-synuclein incubated with dopamine (αSN53o, ^+^<0.05, ^++^<0.01, One way ANOVA), than in neurons with Control at 0.3, 0.4, and 0.5 nA current steps. (**d**) Mean spike frequencies elicited by varying steps of depolarizing current in neurons of layer V in frontal cortex. The frequency was significantly lower in neurons injected with αSNo than in neurons with Control at 0.2, 0.3, 0.4, and 0.5 nA current steps. *<0.05, **<0.01 (One way ANOVA) (**e**). Numeric data of average spike frequencies elicited by 0.1–0.5 nA current steps in neurons of layer II/III (**c**) and V (**d**). (**f**) Resting membrane potential (RMP), spike half-width, and medium afterhyperpolarization (mAHP) in pyramidal neurons of layer II/III and V from frontal cortex slices.

To clarify the pathophysiological changes in neuronal activity induced by α-synuclein oligomers, whole-cell recordings were obtained from pyramidal neurons located in slices of the mouse frontal cortex, with the solutions with or without α-synuclein oligomers being infused intracellularly from a patch pipette. We prepared several kinds of solutions with α-synuclein, as described in previous reports^18,19^, and analyzed the molecular state of α-synuclein solutions by immunoblotting (IB) using anti-α-synuclein antibodies (Fig. 1a). Regarding the solutions with wild-type recombinant α-synuclein, higher-order oligomers were detected only in the solution containing α-synuclein incubated with dopamine for 3 days (Wild type, *DA*); they were not detected in solutions containing α-synuclein incubated without dopamine for 3 days (Wild type, 72 *h*) or without incubation (Wild type, 0 *h*). With respect to A53T mutant recombinant α-synuclein, higher-order oligomers were observed only in the solution containing A53T mutant α-synuclein incubated with dopamine for 3 days (A53T, *DA*), not in that containing α-synuclein incubated without dopamine for 3 days (A53T, 72 *h*) or without incubation (A53T, 0 *h*). By contrast, fibrillary states of A53T mutant α-synuclein were commonly seen in these three conditions.

On the basis of these observations, we elected to use the following for the electrophysiological comparison: (1) α-synuclein incubated with dopamine at 37 °C for 3 days including higher-order WT or A53T oligomers (αSNo or αSN53o), (2) WT or A53T α-synuclein incubated without dopamine for 3 days, and free of higher order oligomers (αSN or αSN53), or (3) the solution without α-synuclein (DA or Control). After filtering to remove α-synuclein fibrils, the pipette solutions with α-synuclein contained soluble monomers and oligomers. Under the application of these pipette solutions, 300-ms-long depolarizing currents were injected through the patch pipette to elicit the spikes (Fig. 1b, bottom). Neurons of layer II/III injected with αSNo, but not with αSN or DA, exhibited more spike frequency adaptations, prolonged interspike intervals, and reduced spike frequency compared with Control neurons (Fig. 1b). For each current intensity of 0.5 nA, the averaged spike frequency in αSNo-injected neurons (28.6 ± 0.6 Hz; n = 7) was significantly smaller than that in Control neurons (39.6 ± 1.0 Hz, *p* < 0.001; n = 9; Fig. 1c,e). The spike frequency in the αSN53o-injected neurons (31.9 ± 1.0 Hz at 0.5 nA; n = 9) was also significantly decreased when compared with Control neurons (*p* = 0.002; Fig. 1c,e). In DA-injected neurons (39.4 ± 1.8 Hz; n = 6), αSN-injected neurons (39.2 ± 1.4 Hz; n = 8), and αSN53-injected neurons (40.8 ± 2.2 Hz; n = 8), the averaged spike frequency was at the same level as Control neurons (Fig. 1c,e). With respect to layer V, the spike frequency for current intensity of 0.5 nA in the αSNo-injected neurons (28.6 ± 1.0 Hz; n = 7), but not in DA-injected neurons (41.0 ± 1.9 Hz; n = 7) and αSN-injected neurons (37.6 ± 1.6 Hz; n = 7), was significantly reduced in comparison with that in Control neurons (38.9 ± 2.9 Hz, *p* = 0.003; n = 6; Fig. 1d,e). In both layer II/III and V, αSNo and αSN53o regulated multiple spike firing in a spike frequency-dependent manner. The averaged spike frequencies in neurons injected with αS oligomer-containing solution, were significantly reduced for current intensities of 0.3 nA and more in layer II/III, and of 0.2 nA and more in layer V (Fig. 1c–e). By contrast, there were no significant between-group differences in RMP and single spike properties such as spike half-width, and medium afterhyperpolarization (mAHP; Fig. 1f). It is thus demonstrated that multiple spikes, but not a single spike, contributed to the αSNo-mediated regulation of spike firing; this strongly suggests that αSNo prolongs the interspike interval and enhances AHP, which is the basis of spike-frequency adaptation^20^.

**Figure 2:**
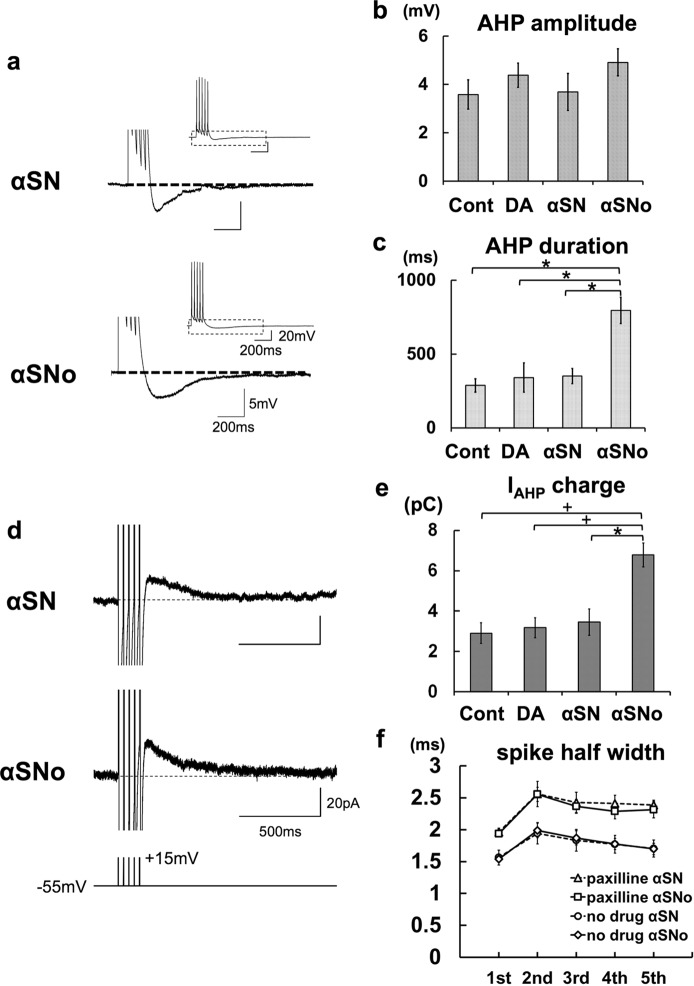
αSNo prolonged the duration of AHP following a train of spikes and increased AHP current charge in comparison with αSN. **(a**) AHP after a train of five spikes at 30 Hz. In neurons with αSNo, AHP duration, but not AHP amplitude, was increased in comparison with neurons with αSN. Calibration: 200 ms, 5 mV. Inset, Overall view of a train of five spikes and the following AHP. Calibration: 200 ms, 20 mV. (**b**,**c**) Summary diagrams demonstrating average AHP amplitude (B) and average AHP duration (C). Injection of αSNo enhanced AHP duration. *p < 0.01 (One way ANOVA). (**d**) I_AHP_ recorded in voltage clamp mode. I_AHP_ were elicited after five brief step depolarization pulses (bottom) that would produce AHP under current clamp. I_AHP_ were integrated to calculate the I_AHP_ charge representing the AHP. Calibration: 500 ms, 20 pA. (**e**) Summary diagram demonstrating the average I_AHP_ charge. The infusion of αSNo significantly augmented the I_AHP_ charge in comparison with αSN, DA, and Control. ^+^*p* < 0.01, ^*^p < 0.02, (One way ANOVA). (**f**) Averaged spike half-width during the five-spike train with no blocker (circle, αSN; diamond, αSNo), and with paxilline (triangle, αSN; square, αSNo).

To test this idea further, we measured AHP after a train of five spikes in pyramidal neurons with αSN or αSNo, and found that α-synuclein oligomers significantly prolonged the duration of AHP following a train of five spikes. The AHP duration was significantly lengthened in αSNo-applied neurons (795 ± 89 ms; n = 6) in comparison with αSN-applied neurons (351 ± 52 ms, *p* = 0.011; n = 6), DA-applied neurons (341 ± 98 ms, *p* = 0.028; n = 6), and Control neurons (288 ± 45 ms, *p* = 0.005; n = 7; Fig. 2a,c). In contrast to AHP duration, the amplitude of the AHP in the αSNo-applied neurons (4.9 ± 0.6 mV) was at the same level as that in the αSN-applied neurons (3.7 ± 0.8 mV), DA-applied neurons (4.4 ± 0.5 mV), and Control neurons (3.6 ± 0.6 mV; Fig. 2a,b). This enhancement of AHP duration gave the αSNo-applied neurons the augmentation of AHP current charge (I_AHP_ charge) observed in voltage clamp mode^20,21^. I_AHP_ charge was significantly increased by the infusion of αSNo (6.8 ± 0.6 pC, n = 5), in comparison with αSN (3.5 ± 0.7 pC, *p* = 0.014, n = 6), DA (3.2 ± 0.5 pC, *p* = 0.009, n = 6), and the Control (2.9 ± 0.5 pC, *p* = 0.004, n = 6; Fig. 2d,e). To the contrary, the spike half-width during a train of five spikes was not affected by the application of αSNo, regardless of the presence or absence of BK channel antagonist paxilline (Fig. 2f). At the fifth spike, the spike half width in the αSNo-injected neurons was 1.70 ± 0.09 ms (n = 6), the same level as that in the αSN-injected neurons (1.70 ± 0.09 ms, n = 6), with this being the case even under the application of paxilline (αSNo, 2.31 ± 0.13 ms, n = 5, *vs* αSN, 2.39 ± 0.08 ms, n = 5; Fig. 2f). This suggests that the involvement of BK-type Ca^2+^-activated K^+^ channel (BK channel) with αSNo-mediated spike reduction is unlikely. These results raise the possibility that α-synuclein oligomers enhance the spike-induced Ca^2+^ transient in neurons via Ca^2+^ influx from VDCC, thereby opening SK-type Ca^2+^-activated K^+^ channel (SK channel) for longer and increasing the duration of AHP in an activity-dependent and Ca^2+^-dependent manner^20,21^.

### α-Synuclein oligomers prolonged AHP by spike-induced Ca^2+^ release from IP3 receptor coupled with L-VDCC and SK channel

**Figure 3:**
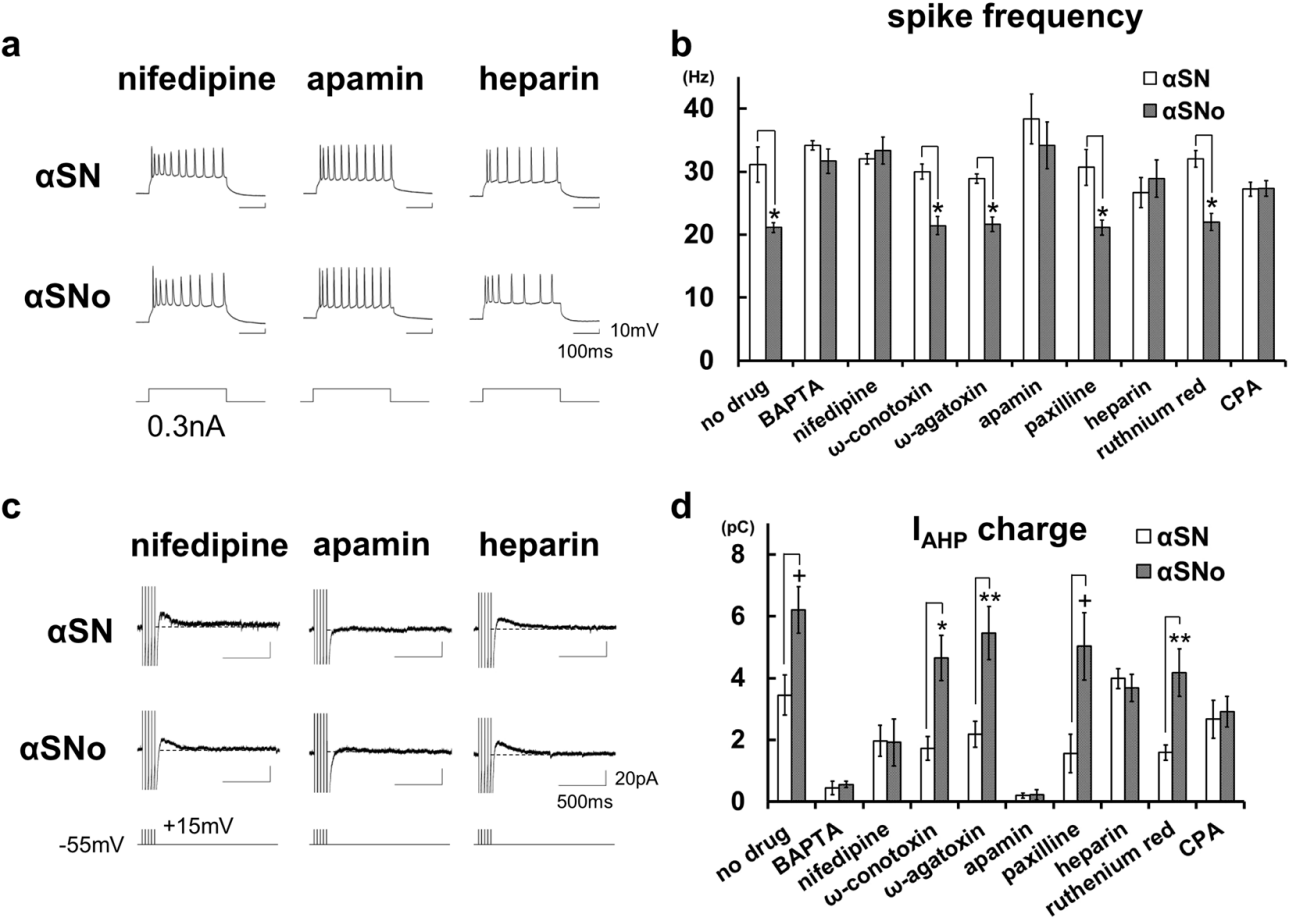
α-Synuclein oligomers prolonged AHP by spike-induced Ca^2+^ release from IP_3_ receptor coupled with L-VDCC and SK channel. **(a**) Specimen recordings of action potentials during positive current pulses (300 ms, 0.3 nA) in neurons with αSN or αSNo under the application of the L-VDCC blocker nifedipine (10 μM), the SK channel blocker apamin (100 nM) and the IP_3_R blocker heparin. These blockers canceled αSNo-mediated reduction of spike frequency. Scale bars, 100 ms and 10 mV. (**b**) Mean spike frequencies during 0.3 nA positive current steps under the application of various blockers of channels or receptors controlling intracellular Ca^2+^ dynamics. *p < 0.01 (*t*-tests) (**c**). Specimen recordings of I_AHP_ in neurons with αSN or αSNo under the application of nifedipine, apamin, and heparin. These blockers canceled αSNo-mediated enhancement of I_AHP_ charge. Scale bars, 500 ms and 20 pA. (**d**) Summary diagram demonstrating the mean I_AHP_ charge under the application of various blockers, each with αSN or αSNo. In B and D, nifedipine, apamin, Ca^2+^ chelator BAPTA, Ca^2+^ store depletor CPA, and heparin canceled the effect of αSNo. *p < 0.01, **p < 0.02, ^+^*p* < 0.03 (*t*-tests).

Which receptors or channels on the plasma membrane or ER regulating intracellular Ca^2+^ and neuronal excitability are involved in the αSNo-mediated effect? To address this question, the spike frequency and I_AHP_ charge in αSNo-injected or αSN-injected neurons were examined under the application of blockers for the channels or receptors responsible for intraneural Ca^2+^ dynamics (Fig. 3).

The application of BAPTA-AM, a chelating agent of intracellular Ca^2+^, abolished the effect of αSNo on spike frequency (αSNo, 31.7 ± 1.9 Hz, n = 4, *vs* αSN, 34.2 ± 0.8 Hz, n = 4; Fig. 3b), and I_AHP_ charge (αSNo, 0.6 ± 0.1 pC, *vs* αSN, 0.5 ± 0.2 pC; Fig. 3d), confirming that the action of αSNo is dependent on intraneural Ca^2+^. As with the VDCC, application of the L-VDCC blocker nifedipine canceled the modulation of spike frequency and I_AHP_ charge in αSNo-injected neurons (33.3 ± 2.1 Hz and 1.9 ± 0.8 pC, n = 5) maintaining the same level as in αSN-injected neurons (32.0 ± 0.8 Hz and 2.0 ± 0.5 pC, n = 5; Fig. 3a–d). Unlike nifedipine, neither the P/Q-type VDCC blocker ω-conotoxin, nor the N-type blocker ω-agatoxin counteracted the effect of αSNo. With ω-conotoxin, the spike frequency with αSNo was 21.4 ± 1.4 Hz (n = 7), which was significantly smaller (*p* = 0.0008) than with αSN (30.0 ± 1.2 Hz, n = 6), while with ω-agatoxin, the spike frequency with αSNo was 21.7 ± 1.1 Hz (n = 6), which was also significantly lower (*p* = 0.0005) than with αSN (28.9 ± 0.7 Hz, n = 6; Fig. 3b). I_AHP_ charge in αSNo-infused neurons was still significantly enlarged when compared with that in the αSN-infused neurons with ω-conotoxin (αSNo, 4.7 ± 0.7 pC *vs* αSN, 1.7 ± 0.4 pC, *p* = 0.006) or ω-agatoxin (αSNo, 5.5 ± 0.9 pC, *vs* αSN, 2.2 ± 0.4 pC, *p* = 0.011; Fig. 3d).

As for the Ca^2+^-activated K^+^ channel, the SK channel inhibitor apamin abolished the αSNo-induced alterations in spike frequency (αSNo, 34.2 ± 3.7 Hz, n = 4, *vs* αSN, 40.0 ± 2.7 Hz, n = 4), and I_AHP_ charge (αSNo, 0.2 ± 0.2pC *vs* αSN, 0.2 ± 0.1 pC; Fig. 3a–d), but the BK channel inhibitor paxilline failed to block αSNo-mediated alteration of spike frequency (αSNo, 21.1 ± 1.2 Hz, n = 6 *vs* αSN, 31.1 ± 2.6 Hz, n = 5, *p* = 0.009; Fig. 3b) and I_AHP_ charge (αSNo, 5.0 ± 1.1 pC *vs* αSN, 1.6 ± 0.6pC, *p* = 0.025; Fig. 3d).

Surprisingly, IP_3_ receptor (IP_3_R) blocker heparin canceled the αSNo-medicated change in spike firing (αSNo, 28.9 ± 2.9 Hz, n = 6, *vs* αSN, 26.7 ± 2.4 Hz, n = 4; Fig. 3a,b) and I_AHP_ charge (αSNo, 3.7 ± 0.4 pC, *vs* αSN, 4.0 ± 0.3 pC; Fig. 3c,d). By contrast, ruthenium red, which blocks ryanodine receptor and mitochondrial Ca^2+^ uniporter, did not alter the αSNo-mediated actions on spike frequency (αSNo, 22.0 ± 1.3 Hz, n = 5, *vs* αSN, 32.0 ± 1.3 Hz, n = 5, *p* = 0.0008; Fig. 3b), and I_AHP_ charge (αSNo, 4.2 ± 0.8 pC, *vs* αSN, 1.6 ± 0.2 pC, *p* = 0.013; Fig. 3d). This result ruled out the involvement of the ryanodine receptor on the ER or the mitochondrial Ca^2+^ uniporter in αSNo-mediated action. Moreover, the ER Ca^2+^ store depletor CPA abolished the αSNo-mediated effects on spike frequency (αSNo, 27.3 ± 1.3 Hz, n = 5, *vs* αSN, 27.2 ± 1.1 Hz, n = 6; Fig. 3b) and I_AHP_ charge (αSNo, 2.9 ± 0.5 pC, *vs* αSN, 2.7 ± 0.6 pC; Fig. 3d), confirming the involvement of Ca^2+^ release from ER in αSNo action and ruling out αSNo-mediated elevation of spike-induced Ca^2+^ influx through VDCC.

Previous studies have established that a Ca^2+^-dependent functional triad consisting of VDCC, IP_3_R and SK channel is linked to spike-triggered Ca^2+^ inflow and Ca^2+^ release from IP_3_R in neurons of the neocortex and amygdala^12–14,21–24^. Therefore, our findings strongly suggest that, via this channel coupling, α-synuclein oligomers mediate Ca^2+^-induced Ca^2+^ release (CICR) from IP_3_R, which are triggered by Ca^2+^ influx via L-VDCC during multiple spikes, followed by the elongation of SK channel opening, the prolongation of I_AHP_, and reductions in spike frequency. Consequently, in neocortical pyramidal neurons, we can detect the occurrence of this mode of CICR by observing the enlargement of I_AHP_ charge and the reduction in spike frequency.

### α-Synuclein oligomers target the regulation of IP_3_R gating and mediate an aberrant form of CICR from IP_3_R during multiple spikes

Which player is the direct target of αSNo mediation of CICR from IP_3_R? IP_3_R has two separate binding sites for Ca^2+^ and IP_3_, with these being regulated allosterically by these two ligands, with binding of one ligand facilitating additional binding of the other^25,26^. Under this positively cooperative mechanism, IP_3_R responds to the increase in neuronal cytosolic Ca^2+^ and IP_3_, and effectively opens, releasing Ca^2+^ from the ER in an activity-dependent manner^12–14,21,27,28^. Accordingly, there are two candidates for the target mechanism by which αSNo causes CICR from IP_3_R: (1) the elevation of IP_3_ turnover; (2) the regulation of IP_3_R gating.

**Figure 4:**
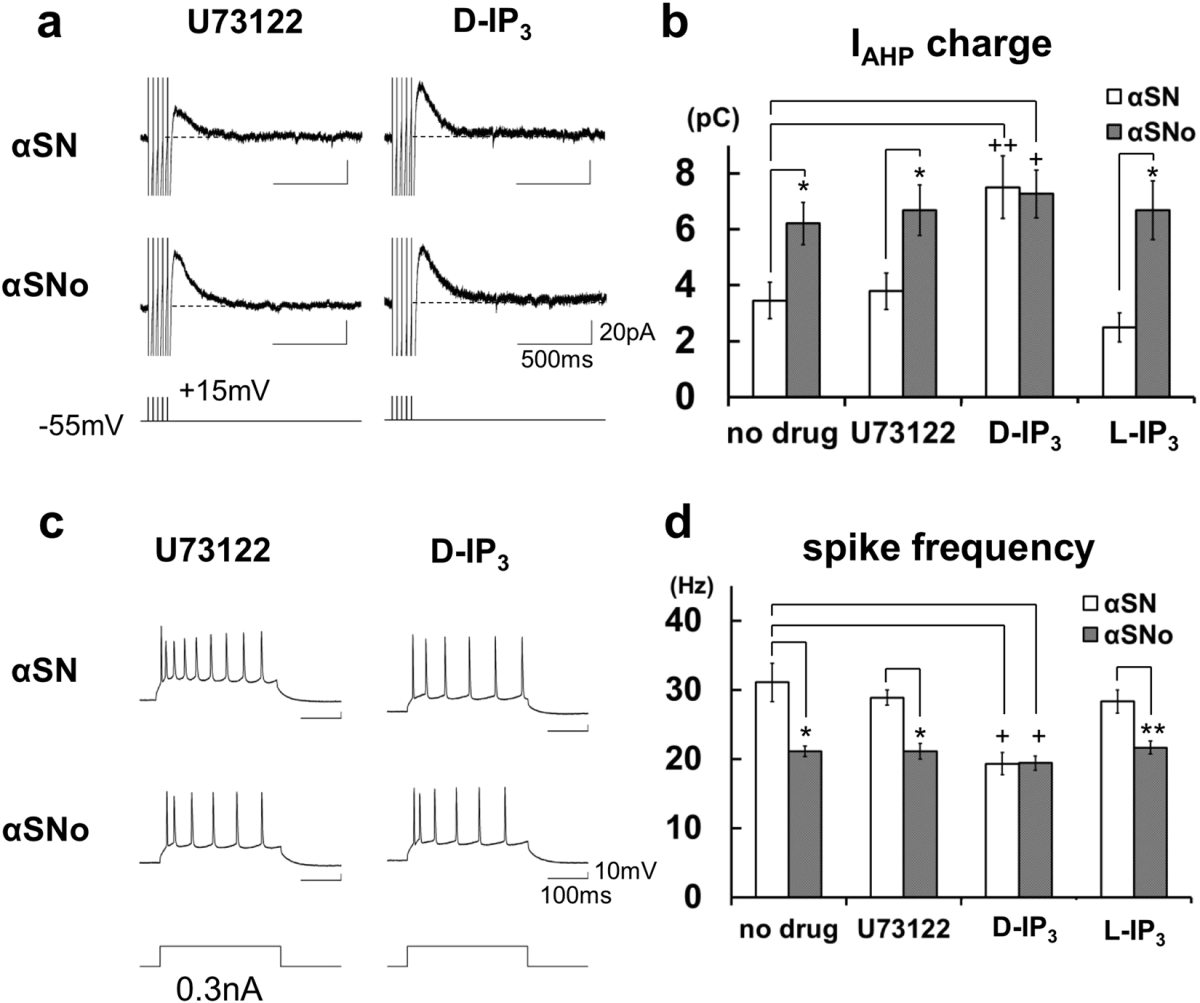
α-Synuclein oligomers target the regulation of IP_3_R and mediate an aberrant form of CICR from IP_3_R during multiple spikes. (**a**) Specimen recordings of I_AHP_ in neurons with αSN or αSNo under the application of D-IP_3_ or U73122. Scale bars, 500 ms and 20 pA. (**b**) Summary diagram demonstrating the mean I_AHP_ charge under the application of D-IP_3_, L-IP_3_, or U73122, each with αSN or αSNo. D-IP_3_, but not U73122 or L-IP_3_, mimicked and occluded αSNo-mediated enhancement of I_AHP_ charge. *p < 0.03 (αSNo *vs* αSN, *t*-tests), ^+^*p* < 0.01, ^++^*p* < 0.02 (D-IP_3_ *vs* no drug, αSN, *t*-tests) (**c**). Specimen recordings of action potentials during the positive current pulse in neurons with αSN or αSNo under the application of D-IP_3_ or U73122. Scale bars, 100 ms and 10 mV. (**d**) Mean spike frequency during current steps under the application of D-IP_3_, L-IP_3_ or U73122. D-IP_3_, but not U73122 or L-IP_3_, mimicked and occluded αSNo-mediated reduction of spike frequency. For the ‘no drug’ example in (**b**,**d**), the same data as shown in Fig. 3 are reproduced for clarity. *p < 0.01, **p < 0.02 (αSNo *vs* αSN, *t*-tests), ^+^*p* < 0.01 (D-IP_3_ *vs* no drug, αSN, *t*-tests).

The first possibility was tested under the application of the phospholipase C (PLC) blocker U73122, which inhibits the hydrolysis of phosphatidylinositol to IP_3_^15,17^. This agent did not block αSNo-induced alteration of I_AHP_ charge (αSNo, 6.7 ± 0.9 pC, n = 6, *vs* αSN, 3.8 ± 0.7 pC, *p* = 0.029, n = 6; Fig. 4a,b) or spike firing rate (αSNo, 21.1 ± 1.1 Hz, *vs* αSN, 28.9 ± 1.1 Hz, *p* = 0.0006; Fig. 4c,d), which preclude the αSNo-mediated enhancement of IP_3_ production.

αSNo can directly upregulate IP_3_R and cause aberrant CICR from IP_3_R without elevating IP_3_ turnover during repetitive spikes, which would not take place under physiological conditions in neocortical pyramidal neuron. In this scenario, intracellular application of IP_3_ will mimic and occlude the action of αSNo. Indeed, I_AHP_ charge and the spike frequency in neurons with D-IP_3_ and αSN (7.5 ± 1.1 pC and 19.3 ± 1.6 Hz, n = 5) were at the same level as those in neurons with D-IP_3_ and αSNo (7.3 ± 0.8 pC and 19.4 ± 1.0 Hz, n = 6; Fig. 4a–d) and αSNo alone (6.2 ± 0.7 pC and 21.1 ± 0.8 Hz, n = 6; αSNo, no drug), and were significantly different from those in neurons with αSN alone (3.5 ± 0.7 pC, *p* = 0.019, and 31.1 ± 2.8 Hz, n = 6, *p* = 0.005; αSN, no drug). In neurons with D-IP_3_ and αSNo, I_AHP_ charge was also significantly larger (*p* = 0.005), while the spike frequency was significantly smaller (*p* = 0.002) than in those with αSN alone (αSN, no drug). In contrast to D-IP_3_, the application of L-IP_3_, a negative analog of IP_3_, had no effect on the αSNo-mediated alteration of I_AHP_ charge (αSNo, 6.7 ± 1.1 pC, n = 4 *vs* αSN, 2.5 ± 0.5 pC, n = 4, *p* = 0.025; Fig. 4a,b) and spike frequency (αSNo, 21.7 ± 1.0 Hz *vs* αSN, 28.3 ± 1.1 Hz, *p* = 0.018; Fig. 4c,d). In combination, our findings indicate that α-synuclein oligomers target the regulation of IP_3_R gating, and mediate the aberrant form of CICR from IP_3_R during repetitive spikes, without enhancing Ca^2+^ influx or IP_3_ production in neocortical neurons.

### The association of α-synuclein oligomers with CaBP1 allows aberrant CICR from IP_3_R by suppressing CaBP1-mediated inactivation of IP_3_R

The gating of IP_3_R is not only regulated by IP_3_ binding; it is also modulated by Ca^2+^ and a variety of proteins^29,30^. Given that αSNo directly targets IP_3_R gating without enhancing Ca^2+^ influx or IP_3_ turnover, αSNo could be associated with the protein that directly binds and regulates IP_3_R in central neurons. To determine the site of action of αSNo and the mechanism by which αSNo mediates CICR from IP_3_R, we bibliographically searched for a protein that meets the conditions, and focused on Ca^2+^-binding protein 1 (CaBP1) amongst the binding partners of IP_3_R, because CaBP1 is (1) a Ca^2+^-binding protein distributed in the cytosol of rodent and human central neurons^31–33^, (2) a preferential interacting protein with α-synuclein oligomers^34^, and (3) a binding partner and negative regulator of IP_3_R under high intraneural Ca^2+^ by means of Ca^2+^-dependent inactivation^35–37^. If αSNo captures CaBP1 and pulls it away from IP_3_R, thus preventing IP_3_R from Ca^2+^-dependent inactivation, an aberrant CICR from IP_3_R could occur, without reinforcing Ca^2+^ influx or cytosolic IP_3_ level.

**Figure 5:**
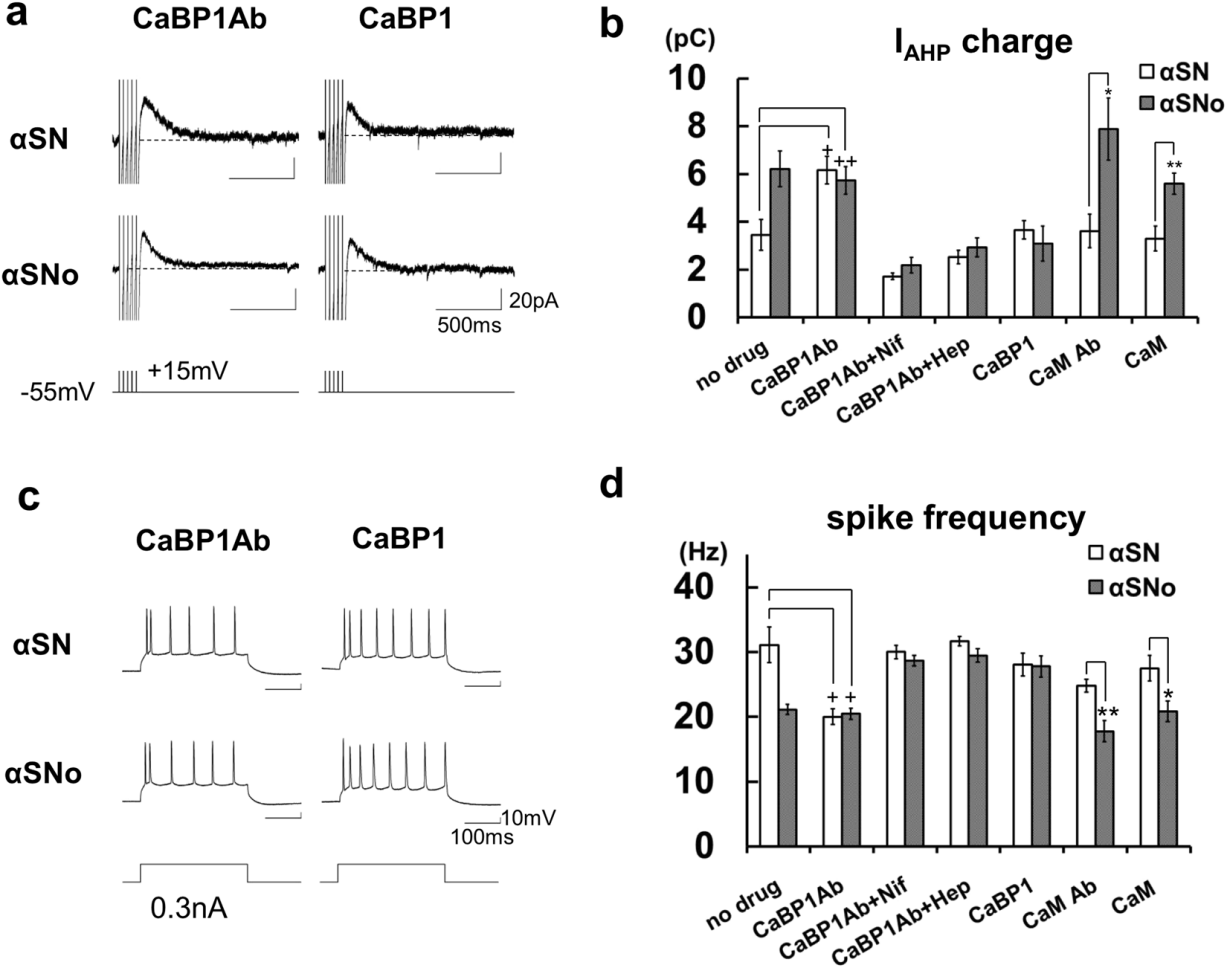
α-Synuclein oligomers suppressed CaBP1-induced inactivation of IP_3_R and triggered spike-induced CICR from IP_3_R. A. (**a**) Specimen recordings of I_AHP_ in neurons with αSN or αSNo under the injection of CaBP1 Ab or CaBP1. Scale bars, 500 ms and 20 pA. (**b**) Summary diagram demonstrating average I_AHP_ charge under the infusion of CaBP1 Ab, CaBP1, calmodulin (CaM) Ab, or CaM, each with αSN or αSNo. *p < 0.01, **p < 0.02 (αSN *vs* αSNo, *t*-tests), ^+^*p* < 0.01, ^++^*p* < 0.02 (CaBP1 Ab *vs* no drug, αSN, *t*-tests) (**c**). Specimen recordings of action po*t*entials during positive current pulse in neurons with αSN or αSNo under the injection of CaBP1 Ab or CaBP1. Scale bars, 100 ms and 10 mV. (**d**) Average spike frequency during current steps under the infusion of CaBP1 Ab, CaBP1, CaM Ab, or CaM, each with αSN or αSNo. For the ‘no drug’ example in (**b**,**d**), the same data as shown in Fig. 3 are reproduced for clarity. *p < 0.01, **p < 0.02 (αSNo *vs* αSN, *t*-tests), ^+^*p* < 0.01 (CaBP1 Ab *vs* no drug, αSN, *t*-tests).

To test this hypothesis, the effects of CaBP1 antibody (Ab) and CaBP1 on αSNo-mediated change were tested. The intracellular co-application of αSN and CaBP1 Ab significantly increased I_AHP_ charge (6.2 ± 0.6 pC, n = 6; Fig. 5a,b) and reduced spike frequency (20.0 ± 1.2 Hz; Fig. 5c,d) in comparison with αSN (I_AHP_ charge, *p* = 0.009, spike frequency, *p* = 0.006; αSN, no drug), and to the same extent as αSNo (αSNo, no drug). The combined infusion of αSNo and CaBP1 Ab exhibited the occlusion of these αSNo-mediated effects (I_AHP_ charge; 5.7 ± 0.6 pC, spike frequency; 20.5 ± 0.9 Hz, n = 7; Fig. 5a–d). In neurons with CaBP1 Ab and αSNo, the I_AHP_ charge was also significantly larger (*p* = 0.019) and spike frequencies significantly smaller (*p* = 0.002) than in those with αSN alone (αSN, no drug).

**Figure 6:**
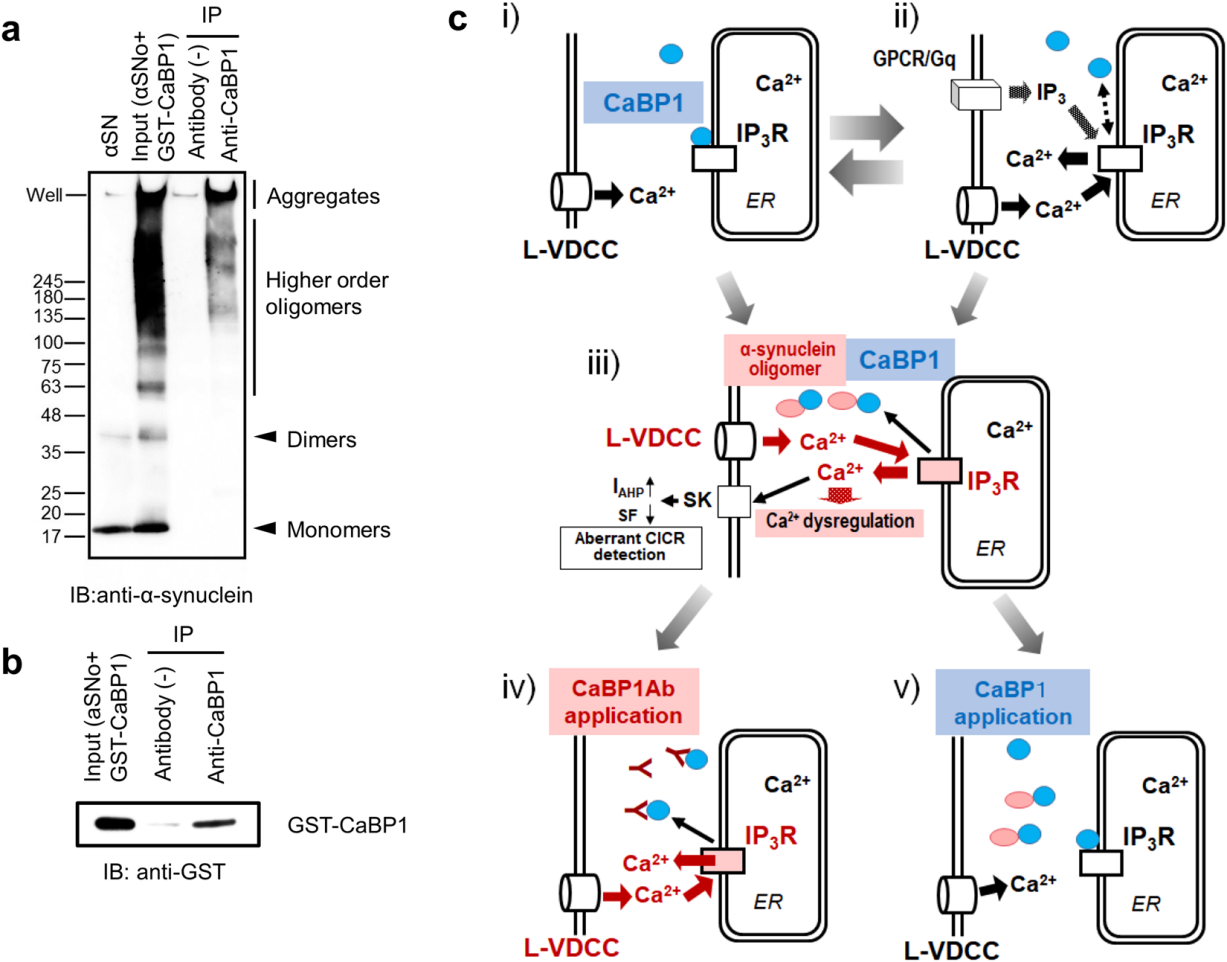
The direct association of α-synuclein oligomers with CaBP1 triggers aberrant CICR from IP_3_R during multiple spikes. (**a**) An immunoprecipitation (IP) experiment. The input solution containing αSNo and GST-CaBP1 was immunoprecipitated with anti-CaBP1 antibody followed by immunoblotting (IB) with antibodies against α-synuclein. (**b**) The input solution containing αSNo and GST-CaBP1 was immunoprecipitated with anti-CaBP1 antibody followed by IB with antibodies against anti-GST. (**c**) Schematic diagram showing the proposed mechanism for α-synuclein oligomer-mediated aberrant CICR from IP_3_R during multiple spikes: (i) Endogenous CaBP1 (blue circle) maintains Ca^2+^-dependent inactivation of IP_3_R (white rectangle). (ii) IP_3_ elevation controlled by finely tuned neurotransmission or neuromodulation is necessary for physiological CICR from IP_3_R. GPCR/Gq: G-protein-coupled receptor/Gq protein. (iii) Intracellular α-synuclein oligomer (red ellipse) captures endogenous CaBP1 and allows the aberrant CICR from IP_3_R (red rectangle) independent of IP_3_ turnover, which boosts Ca^2+^ dysregulation and may lead to selective neuronal fragility in oligomeric α-synuclein-bearing neurons. SF: spike frequency. (iv) Applied CaBP1 Ab binds endogenous CaBP1 and induces CICR in a similar manner to α-synuclein oligomer. (v) Applied CaBP1 binds α-synuclein oligomer and blocks oligomeric α-synuclein-mediated CICR.

The application of nifedipine canceled CaBP1 Ab-mediated action. I_AHP_ charge in neurons with αSNo, was 2.2 ± 0.3 pC (n = 5), and was not significantly different from that with αSN (1.7 ± 0.1 pC, n = 5; Fig. 5a,b). The spike frequency in neurons with αSNo (28.7 ± 0.8 Hz, n = 5) did not differ from that in neurons with αSN (30.0 ± 1.1 Hz, n = 5; Fig. 5c,d). Intracellular co-injection of heparin also inhibited the CaBP1 Ab-mediated effect. In neurons with αSNo, I_AHP_ charge was 2.9 ± 0.4 pC (n = 6), which is the same level as in those with αSN (2.5 ± 0.3 pC, n = 6; Fig. 5a,b). Spike frequency in αSNo-infused neurons was 29.4 ± 1.0 Hz (n = 6), which was not significantly different from that in αSN-infused neurons (31.7 ± 0.7 Hz, n = 6; Fig. 5c,d). These results demonstrate that CaBP1 Ab is sufficient to cause CICR from IP_3_R triggered by Ca^2+^ influx via L-VDCC, and mimics and occludes the effect of αSNo (Fig. 6c iv). Furthermore, the enhancement of I_AHP_ charge and the reduction of spike frequency were reversed in neurons with a co-application of αSNo and CaBP1 (3.0 ± 0.7 pC and 27.8 ± 1.6 Hz, n = 7) to the same extent as in neurons co-injected with αSN and CaBP1 (3.7 ± 0.4 pC, n = 7, and 28.1 ± 1.8 Hz; Fig. 5b,d), thus confirming that CaBP1 blocks the action of αSNo (Fig. 6c v). With consideration of these results, αSNo-mediated capture of CaBP1 is necessary and sufficient for the aberrant CICR from IP_3_R that we observed here (Fig. 6c iii).

By contrast, calmodulin, which is another binding partner and regulator of IP_3_R^38,39^, and directly binds αSN^40,41^, failed to counteract αSNo-mediated modulation of I_AHP_ charge (αSNo; 5.6 ± 0.4 pC, n = 6 *vs* αSN; 3.2 ± 0.5 pC, n = 6; *p* = 0.019, Fig. 5b) and spike frequency (αSNo; 20.8 ± 1.6 Hz *vs* αSN; 27.5 ± 2.0 Hz, *p* = 0.007; Fig. 5d). Calmodulin antibodies did not affect αSNo-mediated alteration of I_AHP_ charge (αSNo; 7.9 ± 1.3 pC, n = 5 *vs* αSN; 3.6 ± 0.7 pC, n = 5, *p* = 0.005, unpaired *t*-test; Fig. 5b) and spike frequency (αSNo; 17.8 ± 1.6 Hz, *vs* αSN; 24.8 ± 1.0 Hz, *p* = 0.013; Fig. 5d).

To determine whether higher-order oligomeric α-synuclein actually binds to CaBP1, we conducted an immunoprecipitation (IP)  experiment. The input solution containing αSNo and GST-CaBP1 was immunoprecipitated with anti-CaBP1 antibody, followed by IB with antibodies against α-synuclein (Fig. 6a) and anti-GST (Fig. 6b). This experiment demonstrated that anti-CaBP1 antibodies were sufficient for IP, but too weak to detect CaBP1 for IB; we therefore used antibodies against GST tagging CaBP1, instead of anti-CaBP1 antibodies, for IB. The results shown in Fig. 6a demonstrate that α-synuclein oligomers larger than 100 kDa and aggregates were present in anti-CaBP1-precipitated samples. We also confirmed that CaBP1 was present in the same batch of the precipitated sample (Fig. 6b). These results indicate the direct binding of higher-order α-synuclein oligomers larger than 100 kDa with CaBP1. In combination, our findings demonstrate that the aberrant CICR occurred only by higher order α-synuclein oligomer larger than 100 kDa.

## Discussion

The present study revealed that intracellularly injected α-synuclein oligomers mediate activity-dependent CICR from IP_3_R, as indicated by the resulting prolonged I_AHP_ and decreased spike frequency in neocortical pyramidal neurons. α-Synuclein oligomers capture CaBP1, and prevent IP_3_R from causing Ca^2+^-dependent inactivation during multiple spikes, thereby releasing Ca^2+^ from ER Ca^2+^ store via IP_3_R without increasing Ca^2+^ influx or IP_3_ turnover. This aberrant form of activity-dependent Ca^2+^ release is mediated only by higher order α-synuclein oligomers larger than 100 kDa, but not by α-synuclein species less than 100 kDa.

In previous studies reporting the effect of intraneural α-synuclein on cytoplasmic Ca^2+^ dynamics and neuronal excitability, transgenic α-synuclein mice exhibited augmented long-lasting Ca^2+^ transients in response to repetitive stimulation *in vivo*^42^, and a reduction in neocortical pyramidal cell excitability was observed by injecting α-synuclein oligomers prepared without dopamine^43^. The present study is consistent with these studies and the first to search in detail for the mechanism of intracellular oligomeric α-synuclein modifying Ca^2+^ handling, and to identify the site of action.

The coupling of the spike-induced Ca^2+^ entry via VDCC and CICR from IP_3_R with the enhancement of the SK channel is a well-documented mechanism in the somatodendritic area of neurons in the neocortex and amygdala; it contributes to the regulation of neuronal excitability and synaptic plasticity^12–14,21–24^. In contrast with previous reports emphasizing that the physiological upregulation of IP_3_ turnover finely tuned by synaptic stimulation or neuromodulation, is necessary for spike-induced or IP_3_-induced Ca^2+^ release from IP_3_R in central neurons^12–14,21,22,27,44,45^ (Fig. 6c i,ii), our observation revealed that oligomeric α-synuclein-mediated CICR from IP_3_R was independent of the elevation of IP_3_ production, because the PLC blocker failed to inhibit it (Fig. 4). Such an unusual mode of CICR provoked by highly frequent neuronal activity, independent of IP_3_ turnover, does not usually take place in central neurons, as the regulation of IP_3_R gating displays bell-shaped dependence on cytosolic Ca^2+^ concentration^46^. This mode of CICR can therefore be reasonably considered as pathological, imposing an excess Ca^2+^ burden on neurons (Fig. 6c iii).

The reason why we used dopamine is to obtain stabilized α-synuclein oligomers. Co-existence of α-synuclein with dopamine results in the formation of SDS-resistant stable soluble oligomers due to dopamine quinones, which contribute to the inhibition of fibrillization by stabilizing α-synuclein oligomers^18,19^. However, the present αSNo-mediated action is attributable to α-synuclein oligomers per se, but not to dopamine or dopamine quinones, and the possibility is also excluded that the intracellular presence of dopamine or dopamine quinones may cause some additional artifactual effects on neuronal properties as follows. First, the injection of α-synuclein oligomers produced without dopamine also results in a spike reduction similar to our findings^43^. Second, the application of DA failed to alter neuronal excitability (Fig. 1). Third, the possibility that monomeric α-synuclein-dopamine adducts may be part of the overall effect is unlikely because monomeric α-synuclein fails to bind CABP1 (Fig. 6) and does not mediate the aberrant CICR.

Neuronal Ca^2+^-binding proteins (CaBPs), a sub-branch of the calmodulin superfamily, are Ca^2+^-sensor proteins, and regulate various Ca^2+^ channel targets^39,47^. CaBP1, a splice variant of CaBPs, is distributed in the cytosol of central neurons^31–33^, and is a preferential α-synuclein oligomer interacting protein, as shown by a co-immunoprecipitation study^34^. As CaPB1 has four EF-hand Ca^2+^-binding motifs, and can bind and regulate IP_3_R under high intraneural Ca^2+^ ^35–37^, the inhibition of interaction between CaBP1 and IP_3_R can result in the aberrant activity-dependent CICR from IP_3_R without increasing IP_3_ production (Fig. 6c iii). We identified the target of α-synuclein oligomers as CaBP1 by electrophysiological recordings (Fig. 5a–d), and confirmed the direct association of α-synuclein oligomers greater than 100 KDa and CaBP1 by IP (Fig. 6a).

Previous reports demonstrated that IP_3_ and CaBP1 have opposing effects on the IP_3_R channel under high intracellular Ca^2+^ concentrations^35–37^. IP_3_ disrupts the inter-subunit interaction of IP_3_R and promotes IP_3_R channel opening (Fig. 6c ii), while CaBP1 binds IP_3_R and clamps the inter-subunit interaction of IP_3_R when the IP_3_ level is low, thereby inhibiting IP_3_R channel opening in a Ca^2+^-dependent manner (Fig. 6c i). These mechanisms explain why either IP_3_ or CaBP1 Ab mimics and occludes and either heparin or CaBP1 blocks the effect of αSNo in our experiment (Figs 4, 5 and 6c). They indicate that oligomeric α-synuclein-mediated deprivation of CaBP1-medicated regulation of IP_3_R, but not the Ca^2+^ buffering effect of CaBP1, is responsible for the aberrant CICR from IP_3_R that we show here.

Although we observed I_AHP_ and spike frequency for detecting the aberrant CICR, SK activation is one of the actions mediated by the aberrant CICR, and how Ca^2+^ dysregulation by this aberrant CICR contributes to distinct pathophysiological mechanism, remained to be studied. Intriguingly, immunohistochemical studies reveal the expression level of CaBP1 in SNc neurons, most fragile in LBD, is lowest amongst central neurons^31,33^. Moreover, the aberrant CICR from IP_3_R propagates as a Ca^2+^ wave along the ER via IP_3_Rs and ryanodine receptors throughout the somatodendritic portion and the nucleus^22,25,26,48^. A sporadic PD risk gene BST1 encodes cyclic ADP-ribose hydrolase 2, synthesizing cyclic ADP-ribose, a ryanodine receptor agonist^49^. Variant BST1 may disturb normal channel function of ryanodine receptor, another Ca^2+^ release channel from ER, and enhance the propagation of dysregulated Ca^2+^ wave mediated by the aberrant CICR via IP_3_R. Chronic occurrence of this propagated aberrant CICR may increase a risk of activity-dependent distinct Ca^2+^ dysregulation and may lead to neuronal fragility in oligomeric α-synuclein-bearing neurons^6,7,50–53^, although this remains to be examined.

## Material and Methods

### Slice preparations

All experiments were performed in accordance with the guiding principle of the Physiological Society of Japan and with the approval of the Animal Care Committee of Utano National Hospital. C57BL/6 wild mice (P20–50) of either sex were deeply anesthetized with isoflurane and decapitated. The brain was dissected out and immersed in bathing medium (pH 7.4; 2–5 °C) containing (in mM) 124 NaCl, 3.3 KCl, 1.3 NaH_2_PO_4_, 26 NaHCO_3_, 2.5 CaCl_2_, 2.0 MgSO_4_, and 20 glucose. Frontal cortex slices of 220 μm were prepared with a microslicer (Linearslicer PRO-7, Dosaka, Kyoto, Japan).

### Electrophysiological recordings

Electrophysiological recordings were performed as described previously^15,16^. Briefly, slices were placed in a recording chamber on the stage of an upright microscope (BHWI; Olympus, Tokyo, Japan) with a 40× water-immersion objective (LUMPlan FI/IR 40x/0.80 W). The chamber was continuously perfused with bathing medium (25 °C) bubbled with a mixture of 95% O_2_ and 5% CO_2_. For recording, patch pipettes (resistance, 5–10 MΩ) filled with a solution (pH 7.3) containing (in mM) 7 KCl, 144 K-gluconate, 10 KOH, 10 HEPES, 4 Na_2_ATP, and 0.4 Na_2_GTP were used. Whole-cell recordings were made at the soma from layer II/III or V pyramidal neurons in frontal cortex slices. Capacitance was compensated to 70–80%. Neurons that had sufficiently negative resting membrane potentials (RMPs; more negative than −60 mV) without spontaneous action potentials were selected.

Recombinant α-synuclein [Wild type (WT) or A53T mutant (A53T), Sigma] was dissolved with sterile water (10 µM) and co-incubated with 100 µM dopamine hydrochloride (Sigma) at 37 ^o^C for 3 days (αSNo or αSN53o). For comparison, 10 µM recombinant α-synuclein (WT or A53T) solution was incubated without dopamine at 37 °C for 3 days (αSN or αSN53). The αSNo or αSN53o included higher-order oligomers of α-synuclein, which were absent in αSN or αSN53 (Fig. 1a). Dopamine (100 µM) without α-synuclein was also prepared, and incubated at 37 °C for 3 days (DA). All these solutions were filtered through a PVDF filter (millex-HV SLHV004SL; Merck Millipore, Darmstadt, Germany), which removed α-synuclein aggregates, including fibrils more than 0.45 μm. These solutions were diluted for the patch pipette internal solution, so that the final concentration of α-synuclein and DA were 1 µM and 10 µM, respectively. Present experiments used the solutions containing α-synuclein oligomers made without phosphate buffered salts (PBS), which are generally used to get a better control of ionic strength and PH^54^, to avoid the possibility that PBS could unexpectedly affect spike property and Ca^2+^ dynamics when injected intracellularly.

These solutions or the vehicle solution (Control) were distributed into the cell by diffusion (infusion) for at least 5 min after whole-cell break in, and before the recording session commenced. Membrane potentials were recorded in the current clamp mode (Axopatch 200B; Axon Instruments, CA, USA) and digitized at 10 kHz (Digidata 1440 and pCLAMP10, Axon Instruments). Depolarizing currents (0.1–0.5 nA for 300 ms) were injected through the patch pipette to assess the membrane excitability of the recorded neurons. A single action potential or trains of five spikes at 30 Hz were evoked by a 3–5 ms depolarization current pulse (0.7–0.9 nA) or five depolarization current pulses, respectively. The width of the evoked spikes was measured at 50% of the peak. The afterhyperpolarization (AHP) current (I_AHP_) was recorded in voltage clamp mode. I_AHP_ charge was defined as the charge transfer carried by the current elicited by five depolarization pulses that would produce AHP under current clamp. I_AHP_ were integrated after these step depolarization pulses to calculate the charge transfer (I_AHP_ charge) representing the AHP.

### Drugs used

Depending on the purpose of the electrophysiological experiments, the chemicals applied via the recording solution included nifedipine (10 μM), ω-conotoxin (1 μM), ω-agatoxin (50 nM), apamin (100 nM), paxilline (10 μM), cyclopiazonic acid (CPA; 30 μM; all purchased from Alomone Labs, Jerusalem, Israel), BAPTA-AM (1 0 μM; Sigma), or U73122 (4 μM; Sigma). The pipette solution also included heparin (low molecular weight; 4 mg/ml; MP Biomedicals), ruthenium red, D-IP_3_, L-IP_3_ (100 μM; Alomone Labs), glutathione S-transferase (GST)-CaBP1 (16 nM; Abnova Corp.), calmodulin (3 μM; BioVision Inc.), CaBP1 antibody (10 μg/ml; Novus Biologicals), and calmodulin antibody (10 μg/ml; Novus Biologicals).

### Western blotting

Recombinant α-synuclein solution was mixed in equal amounts with a sample-loading buffer. After denaturation by boiling at 100 °C for 5 min, samples were loaded onto a 4‒20% SDS-polyacrylamide gel, separated electrophoretically, and transferred to a polyvinylidene fluoride membrane (Millipore, Bedford, MA, USA). Each lane contains the same amount of protein (1.5 ug). After blocking with non-fatty milk, the membrane was incubated with anti-α-synuclein antibody (Sigma) and horseradish peroxidase-conjugated secondary antibody (GE Healthcare, Little Chalfont, UK). Immunodetection was performed using the ECL Western blotting detection system (GE Healthcare).

### Immunoprecipitation (IP)

GST-CaBP1 protein (100 ng/µL) was added in equal amounts to αSNo containing wild type recombinant α-synuclein (10 µM) and dopamine (100 µM) and incubated for 1 h at 37 °C. The mixture was incubated with or without anti-CaBP1 antibody (Sigma) for 1 h at 37 °C followed by incubation with Protein G sepharose 4 Fast Flow (GE Healthcare) for 1 h at 4 °C with gentle shaking. The beads were precipitated by centrifugation and washed four times with an excess volume of Tris-buffered saline containing 0.1% Triton X-100. Proteins bound to beads were eluted by boiling in a sample-loading buffer. Western blotting was performed as described above, except that anti-α-synuclein antibody (Sigma) and anti-GST antibody (Nacalai Tesque, Kyoto, Japan) were used.

### Experimental design and statistical analysis

As was the case for the electrophysiological recordings, experimental data were obtained from four to nine cells in neocortical slices of brains from mice of either sex. Data are expressed as mean ± SEM. “One way ANOVA followed” by post hoc Turkey HSD tests or Games-Howell tests, and paired and unpaired *t*-tests were used for statistics (SPSS v22, Japan IBM Ltd, Tokyo, Japan).
